# Multifaceted roles of CCAR family proteins in the DNA damage response and cancer

**DOI:** 10.1038/s12276-023-01139-1

**Published:** 2024-01-04

**Authors:** D. Lugano, L. Barrett, S. D. Westerheide, Y. Kee

**Affiliations:** 1 Department of Molecular Biosciences, College of Arts and Sciences, University of South Florida, Tampa, FL 33647 USA; 2 Department of New Biology, Daegu Gyeongbuk Institute of Science and Technology (DGIST), 333 Techno-Joongang-daero, Dalseong-gun, Daegu, 42988 Republic of Korea

**Keywords:** Cancer genetics, Cell signalling

## Abstract

The cell cycle apoptosis regulator (CCAR) family of proteins consists of two proteins, CCAR1 and CCAR2, that play a variety of roles in cellular physiology and pathology. These multidomain proteins are able to perform multiple interactions and functions, playing roles in processes such as stress responses, metabolism, and the DNA damage response. The evolutionary conservation of CCAR family proteins allows their study in model organisms such as *Caenorhabditis elegans*, where a role for CCAR in aging was revealed. This review particularly highlights the multifaceted roles of CCAR family proteins and their implications in the DNA damage response and in cancer biology.

## CCAR proteins: key players in DNA damage response and cancer biology

Cell Division Cycle Apoptosis Regulator (CCAR, proteins that regulate cell division and death) proteins play various roles in stress response, DNA repair, and longevity regulation. Their absence or overexpression could lead to cancers, with unclear underlying mechanisms. Recent studies explored the roles of CCAR2 protein, using molecular and cellular techniques (methods for studying cells and molecules), which discovered that human CCAR2 directly aids in the repair of DNA double-strand breaks, highlighting its diverse roles in stress responses. Studies also underscored the involvement of CCAR2 in the DNA damage response and repair, apoptosis, transcription, and aging. The findings could help in understanding and treating diseases that harbor alterations in CCAR2 gene.

This summary was initially drafted using artificial intelligence, then revised and fact-checked by the author.

## Introduction

Cell division cycle apoptosis regulator (CCAR) proteins are evolutionarily conserved. Studies have highlighted their versatile roles in RNA regulation, stress responses, longevity regulation, and the DNA damage response. Recent studies further found that human CCAR2 (hCCAR2) directly participates in the repair of DNA double-strand breaks, underscoring its multifaceted roles in stress responses^1^. Deficiencies in these roles are expected to contribute to tumorigenesis. Below, we highlight the multifaceted roles of CCAR family proteins and their implications in cellular stress responses and in cancer biology.

## Structural features of CCAR proteins

**Fig. 1:**
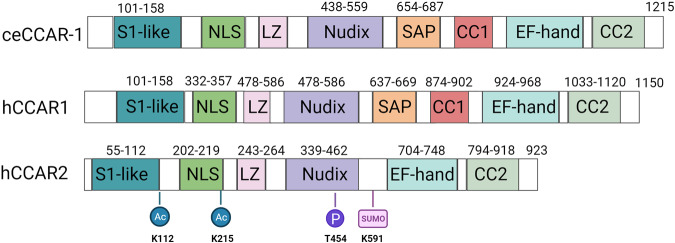
The domain structure of CCAR family members. The domains in *C. elegans* CCAR-1 (*ce*CCAR-1) along with human CCAR1 (hCCAR1) and human CCAR2 (hCCAR2) are shown, along with the amino acid boundaries for each domain. Functionally studied sites of posttranslational modifications, including acetylation (Ac), phosphorylation (P), and sumoylation (Sumo), in hCCAR2 are indicated. The drawing is not to scale.

Key to the dynamic nature of CCAR family proteins is the presence of conserved domains that allow them to interact with several factors^2^. These domains include the S1-like, nuclear localization signal (NLS), leucine zipper (LZ), nudix, EF-hand, SAP, and coiled-coiled domains (Fig. 1). The S1-like domain is homologous to domains possessing RNA-binding capability^2^. A presumed nuclear localization domain (NLS) is immediately downstream of the S1-like domain^3,4^. A leucine zipper (LZ) domain and a Nudix domain are next in the sequence. LZ domains are known to regulate several cellular pathways by diverse mechanisms, including via interactions with NFκB family members^5^. The Nudix domain is predicted to act as a metabolic sensor for CCAR activity via the binding of nicotinamide adenine dinucleotide (NAD + ) metabolites^6^. The C-terminus contains an EF-hand domain and a coiled-coiled domain. The EF-hand domain is an inactive variant of a calcium-dependent regulator of multiple cellular processes^7^, and the coiled-coiled domain is predicted to participate in protein‒protein interactions^2,8^. Some members of the CCAR family possess a SAP domain, utilized for transcriptional regulation, DNA damage repair, RNA processing^2^. This domain is common in DNA damage repair genes such as poly ADP-ribose polymerase 1 (PARP1), X-ray repair cross-complementing protein (Ku70), and restriction site-associated DNA protein 18 (RAD18)^9–11^.

Human CCAR2 (hCCAR2), formerly known as deleted in breast cancer (DBC1), initially gained interest due to its homozygous deletion in breast cancer^2,12–14^. hCCAR2 has a paralog, hCCAR1, which phylogenetic analyses predict to have evolved from a shared ancestor, *Caenorhabditis elegans* CCAR-1 (*ce*CCAR-1), previously known as LST-3^2^. hCCAR2 and *ce*CCAR-1 have 30% amino acid sequence similarity and contain similar domains. All domains of CCAR family proteins are conserved between the two species; however, hCCAR1 and *ce*CCAR-1 contain an SAP domain largely linked to a role in the DNA damage response^2^. CCAR proteins are intrinsically disordered, allowing structural flexibility. In human CCAR2 and hCCAR1, 41% of the residues are disordered, while in *ce*CCAR-1, 61% are disordered^2^. Intrinsically disordered regions are frequent sites of protein interactions and posttranslational modifications.

Various posttranslational modifications, including acetylation, phosphorylation, and SUMOylation, have been identified on hCCAR2, and all can alter its function (Fig. 1). For example, hCCAR2 is phosphorylated by ataxia-telangiectasia mutated (ATM/ATR) at Thr454, which increases sirtuin 1 (SIRT1) binding^15^. hCCAR2 can also be acetylated by the human males absent on the first (hMOF) protein at K112 and K215, leading to the disruption of SIRT1-hCCAR2 binding and increased SIRT1 activity^4^. Additionally, during genotoxic stress, hCCAR2 is sumoylated at K591 by the small ubiquitin-related modifier protein (SUMO2/3) and protein inhibitor of activated STAT3 (PIAS3) E3 ligases^16^. hCCAR sumoylation leads to an increase in the transactivation of p53, which can be reversed by the activity of the desumoylase SENP1^17^. However, much is unknown about the posttranslational modifications on *ce*CCAR-1.

## hCCAR2 regulates SIRT1

**Fig. 2:**
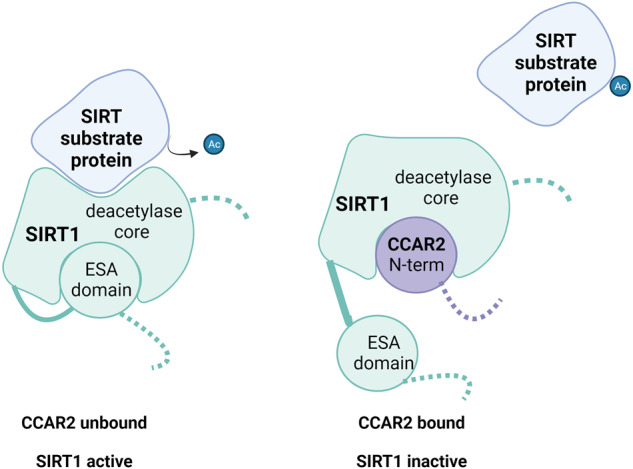
Model of the mechanism of inhibition of SIRT1 by CCAR2. SIRT1 contains a 25-residue domain called the Essential for SIRT1 Activity (ESA) domain. This domain folds back and binds to SIRT1’s deacetylase core to allow the deacetylase activity of SIRT1. hCCAR2 binds to the SIRT1 deacetylase core via an N-terminal domain and inhibits the ESA domain from interacting with the deacetylase core, thus inhibiting SIRT1 activity upon binding.

One of the most extensively studied functions of hCCAR2 is its inhibition of SIRT1, an NAD^+^-dependent deacetylase that regulates various processes, such as apoptosis, stress responses, metabolism, longevity, and cancer-related processes^13,14^. This regulation occurs through transcription factors, including p53, forkhead box gene O (FOXO), heat shock factor 1 (HSF1), and peroxisome proliferator-activated receptor gamma coactivator 1 alpha (PGC1α). hCCAR2 binds to the catalytic domain of SIRT1, forming a stable complex that inhibits SIRT1 deacetylase activity and function both in vivo and in vitro^13^. SIRT1 contains a 25-residue domain called the Essential for SIRT1 Activity (ESA) domain, which must fold back and bind to SIRT1’s deacetylase core to allow its activity. hCCAR2, which also binds to the deacetylase core, inhibits the interaction of the ESA region with the deacetylase core, thus inhibiting SIRT1 activity upon binding^18^ (Fig. 2).

Protein kinase A (PKA) and AMP-activated protein kinase (AMPK) cause the dissociation of hCCAR2 from SIRT1 in an NAD^+^-independent manner^19^. Additionally, hCCAR2 regulates SIRT1 activity by sensing the soluble products or substrates of the NAD^+^-dependent deacetylation reaction^6^. These include soluble ligands with a nucleoside diphosphate moiety, such as ADP-ribose. This binding of ADP-ribose derivatives to the SIRT1-hCCAR2 complex occurs through sensing of SIRT1 by hCCAR2 and the subsequent specific downregulation of SIRT1, a mechanism that is supported by the proximity of SIRT1 to the coiled-coiled domain and inactive Nudix domain in hCCAR2^6^.

## CCAR modulates stress responses

**Fig. 3:**
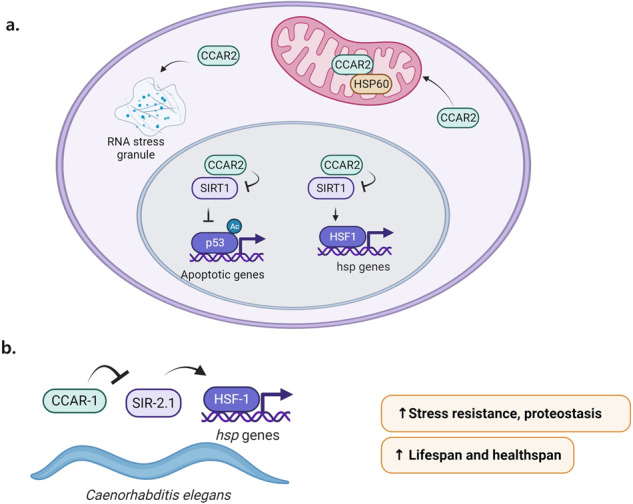
Roles of CCAR proteins in stress responses and aging. **a** hCCAR2 modulates mammalian stress responses. hCCAR2 promotes apoptosis by inhibiting SIRT1 activity and thus increasing the level of the active, acetylated form of p53 and inducing the transcription of apoptotic genes. Conversely, hCCAR2 negatively regulates the HSR in humans by inhibiting SIRT1 and thus increasing HSF1 acetylation, decreasing the binding of HSF1 to its target hsp gene promoters. Upon mitochondrial stress, hCCAR2 localizes to mitochondria, where it binds to HSP60 and promotes cell survival. Additionally, hCCAR1 localizes to and regulates the formation of RNA stress granules where subsets of mRNAs are sequestered after stress for translational silencing. **b** *ce*CCAR-1 modulates *C. elegans* stress responses and lifespan. *ce*CCAR-1 negatively regulates the heat shock response through interaction with SIR-2.1, the worm homolog of SIRT1. Knockdown of *ce*CCAR-1 in *C. elegans* was found to work through SIR‐2.1 to promote stress resistance and proteostasis and to increase the lifespan and healthspan.

The CCAR family has been implicated in a variety of stress responses in cells (Fig. 3). For example, hCCAR2 was found to negatively regulate the heat shock response (HSR) in humans through SIRT1 by decreasing HSF1 binding to the promoters of its target molecular chaperone genes (Fig. 3a)^20^. This pathway is also conserved in *C. elegans*, where *ce*CCAR-1 negatively regulates the heat shock response through interaction with the worm homolog of SIRT1 (SIR-2.1) (Fig. 3b)^21^. Upon examination of *ce*CCAR-1 functions in physiological health, knockdown of *ce*CCAR-1 was found to exert effects through SIR‐2.1 to promote stress resistance and proteostasis and increase the lifespan and healthspan at the whole-organism level^21^ (Fig. 3b). In mammalian cells, hCCAR2 regulates mitochondrial stress through its binding to the mitochondrial heat shock protein HSP60 (Fig. 3a). Upon mitochondrial stress, hCCAR2 localizes to mitochondria, where it binds to HSP60 and promotes cell survival^22^. Additionally, hCCAR1 localizes to and regulates the formation of RNA stress granules, where subsets of mRNAs are sequestered after stress for translational silencing (Fig. 3a)^23^. hCCAR2 also regulates apoptosis through its effects on p53 transcriptional activity (Fig. 3a)^16^.

## Role of CCAR2 in the regulation of the DNA damage response and DNA damage repair

**Fig. 4:**
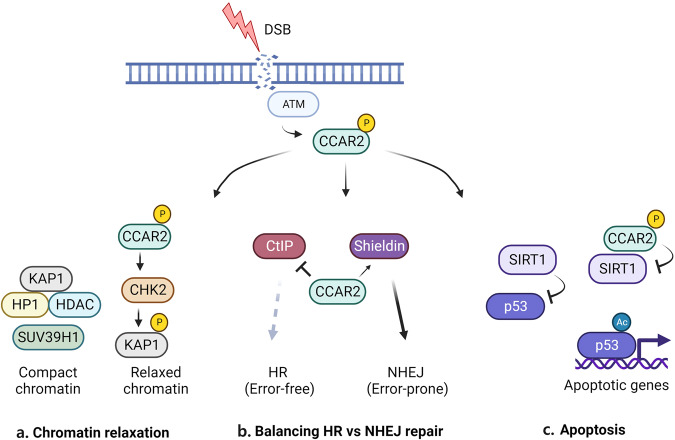
hCCAR2 promotes the DNA damage response and DNA damage repair in multiple ways. **a** hCCAR2 promotes CHK2-induced phosphorylation of KAP1, which may inhibit the recruitment of histone-modifying factors, such as HP1 and SUV39H1 and induce chromatin relaxation. hCCAR2 may directly inhibit SUV39H1 and HDAC3 (not shown). **b** hCCAR2 inhibits CtIP-mediated DSB end resection and HR repair while promoting the 53BP1-RIF1-Shieldin pathway to induce NHEJ repair. **c** hCCAR2 inhibits SIRT1-induced deacetylation of p53, leading to p53 activation and apoptosis. Additionally, hCCAR2 binds and inhibits PARP1, which is involved in various aspects of DNA repair (not shown).

hCCAR2 is a direct target of phosphorylation by ATM, the master kinase that senses DNA damage and activates many downstream targets to allow DNA repair or apoptosis^24,25^. This provides a direct mechanism by which hCCAR2 can mediate the DNA damage response (DDR). Upon phosphorylation by ATM, hCCAR2 regulates many aspects of the DDR, including chromatin relaxation to allow repair factor access, DNA double-strand break (DSB) repair, and apoptosis if the damage is extreme (Fig. 4). While hCCAR2 must interact with SIRT1 to induce apoptosis in response to DNA damage, SIRT1 is dispensable for hCCAR2-mediated modulation of the other aspects of the DDR.

### DNA damage induces CCAR2 in a SIRT-dependent manner, modulating the DDR through PARP1, inducing HSPs, and inducing apoptosis

There are several ways in which hCCAR2 modulates the DDR that are dependent on the hCCAR2-SIRT1 interaction. For instance, hCCAR2 binds to and inhibits PARP1, an enzyme that mediates many aspects of DNA repair, in a SIRT1-dependent manner^26^. PARP1 is recruited to sites of DNA breaks, where it is activated and catalyzes the addition of poly ADP-ribose (PAR) chains on target proteins, including histone H1, histone H2B and PARP1 itself^27^. These PAR chains allow the recruitment of DNA repair proteins, including the MRN complex, which then recruits and activates ATM to initiate the DNA damage response^27^. Interestingly, the ability of hCCAR2 to inhibit PARP1 depends on the binding of NAD^+^ to the Nudix homology domain (NHD) of hCCAR2; this observation identifies a conserved NAD^+^ binding pocket in hCCAR2 regulating NAD^+^ level-dependent DNA repair. This regulation thus links the metabolic regulation of PARP1 to hCCAR2, as the NAD+ level can fluctuate during caloric restriction, aging, and specific disease states^28^.

As discussed earlier, the hCCAR2-SIRT1 interaction leads to SIRT1 inhibition and thus p53 activation. This interaction is regulated by DNA damage stress, which induces p53 activation by maintaining p53 acetylation (Fig. 4c). Indeed, studies found that p53 activation resulting from the hCCAR2-SIRT1 interaction is induced by DNA damage; upon treatment with DNA damaging agents, the hCCAR2-SIRT1 interaction was enhanced by phosphorylation of hCCAR2 at Thr454 by the ATM kinase^24,25^. Blocking hCCAR2 phosphorylation was shown to suppress the hCCAR2-SIRT1 interaction, reduce p53 activation, and suppress apoptosis. This suggests that the hCCAR2-SIRT interaction forms an important axis in the DNA damage response, leading to apoptosis. Interestingly, the hCCAR2-SIRT1 interaction is deregulated in many types of cancers^29,30^, providing cancer cells protection against DNA damage-induced apoptosis. In addition to these SIRT1-dependent roles of hCCAR2 in the DNA damage response, SIRT1-independent roles of CCAR2 have also been described, as discussed below.

### CCAR2 promotes DNA double-strand break repair via nonhomologous end joining, independent of SIRT1

Among the many forms of DNA damage, DNA double-strand breaks (DSBs) are the most dangerous, as unrepaired DSBs can result in large-scale chromosomal translocation, loss of genomic information, and cancer. There are two primary DSB repair pathways in eukaryotic cells: homologous recombination (HR) and nonhomologous end joining (NHEJ)^31,32^. HR is used during the S and G2 phases of the cell cycle, wherein it copies the homologous DNA sequence in sister chromatids (duplicated copies of DNA) to fill in the lost nucleotides at the DNA break site. NHEJ is preferentially used during G1 phase but can be used throughout the cell cycle; in NHEJ, the two broken DNA ends are ligated without a need for the sister chromatid. The usage of HR versus NHEJ must be regulated, as incorrect usage of one versus the other can severely compromise genomic integrity; overuse of HR can lead to hyperrecombination and chromosomal aberrations, while overuse of NHEJ (or its misuse during S or G2 phase) can lead to misjoining of DNA that can result in chromosomal translocations^31–33^. It is no surprise that cells employ sophisticated controls to determine the pathway choice. The current model suggests that “DSB end resection”, wherein the broken DNA is subjected to degradation by a series of nucleases to generate a single-strand DNA overhang, is a decisive step in determining the pathway choice, as the ssDNA becomes a nucleofilament coated by the RAD51 recombinase to initiate HR by invading the sister chromatid.

Various factors have been discovered to regulate or compete to determine the DNA damage repair pathway choice, and recent proteomic analyses implicate hCCAR2 in controlling the activity of some of these factors. Factors promoting HR include breast cancer gene 1 (BRCA1), the MRN complex, and the carboxy-terminal binding protein (CtBP)-interacting protein (CtIP) exonuclease, which promotes DSB end resection and limits NHEJ activity. Factors promoting NHEJ include p53 binding protein (53BP1), Rap1-interacting factor (RIF1), mitotic arrest deficient 2 MAD2L2/REV7, and the Shieldin complex, which have been shown to antagonize end resection, thus shifting the pathway choice toward NHEJ^31,33–37^. A study using a genome-wide RNAi screen to search for new regulators of the pathway choice identified hCCAR2^38^. The results showed that hCCAR2 physically binds to CtIP to limit DSB end resection, leading to HR inhibition. Consequently, hCCAR2 depletion leads to hyperrecombination (Fig. 4b).

The role of hCCAR2 as an HR antagonist is further supported by a recent study in which hCCAR2 was identified as an interactor of REV7 (a subunit of the Shieldin complex) in a proteomic screen^1^. The S1 domain of hCCAR2 was identified as the interaction site. The Shieldin complex shields DSBs against resection, thereby promoting NHEJ. Similar to the knockdown of any of the Shieldin complex components, the knockdown of hCCAR2 restored DSB end resection, RAD51 recruitment to DSBs, and HR activity. hCCAR2 was shown to function downstream of and epistatic to the Shieldin complex in limiting HR while promoting NHEJ (Fig. 4b)^1^. Collectively, these findings show that hCCAR2 is an effector of NHEJ and is a critical factor in maintaining the balance between the HR and NHEJ pathways in DNA repair to preserve genomic integrity.

The regulation of the DSB repair pathway choice by hCCAR2 may have clinical ramifications. Cancer cells with HR deficiency (e.g., with BRCA1/2 mutations) are uniquely sensitive to DNA crosslinking agents and PARP inhibitors, but some of these cells acquire de novo resistance to these drugs due to concomitant loss of the NHEJ pathway. Amplified NHEJ is thought to cause genomic instability and cell death in the BRCA-null background. Knocking out hCCAR2 in BRCA-null cells causes drug resistance^1^, further suggesting that hCCAR2 is a component of the NHEJ pathway. Therefore, cancer patients with BRCA mutations may develop drug resistance partially through the accompanying loss of hCCAR2. Indeed, BRCA1-deficient cancer patients with low hCCAR2 protein levels had lower survival rates than those with higher hCCAR2 levels^1^. Last, hCCAR2 also binds to BRCA1 and inhibits its transcriptional activity^39^. This was shown to reduce the expression of SIRT1. Whether hCCAR2 binding to BRCA1, a key HR repair mediator, has any consequence on HR repair is not known.

### hCCAR2 influences DSB repair by regulating chromatin organization dynamics

DSB repair in heterochromatin requires chromatin relaxation to allow repair factors to access the DNA, and studies suggest that hCCAR2 facilitates this process in a SIRT1-independent manner (Fig. 4a). KRAB-associated protein (KAP1/TRIM28) mediates heterochromatin formation by recruiting histone-modifying enzymes such heterochromatin protein 1 (HP1) and the histone-lysine N-methyltransferase SUV39H1^40^. DSB-induced phosphorylation of KAP1 by ATM and checkpoint kinase 2 (CHK2) is a crucial event in regulating KAP1 binding to histone-modifying proteins, leading to chromatin relaxation, which allows access by DNA repair proteins. A study showed that hCCAR2 promotes CHK2 dimerization, thus leading to the activation and CHK2-induced phosphorylation of KAP1^15^. CHK2 depletion results in defective DSB repair in heterochromatin, and interestingly, this defect is rescued by overexpressing CHK2. In addition, hCCAR2 inhibits the activity of the methyltransferase SUV39H1^41^ and histone deacetylase 3 (HDAC3)^42^, leading to chromatin relaxation.

## Impact of CCAR2 mutations in cancers

Given the roles of CCAR2 in various aspects of the DDR and other cellular processes, it is no surprise that CCAR2 expression is frequently altered in cancers. The role of hCCAR2 in cancer development is not straightforward, as studies have reported conflicting results. hCCAR2, previously called DBC1, was initially found to exhibit homozygous deletion in breast cancer^43^, suggesting a tumor-suppressive role, but later studies found hCCAR2 expression to be increased in various cancer types, suggesting an oncogenic role^44^. hCCAR2 is generally regarded as a proapoptotic factor, but the SIRT1-dependent role of hCCAR2 in cancer development is still controversial. As discussed below, whether it is tumor-suppressive or oncogenic is likely to be dependent on the genetic background or stage of the cancer. For example, the abovementioned roles of hCCAR2 in promoting genomic stability indicate that hCCAR2 is a tumor suppressor that prevents initial cancer development; however, hCCAR2 may be needed to preserve the genomic stability and proliferation of cells in mature tumors.

### Tumor-suppressive roles

**Fig. 5:**
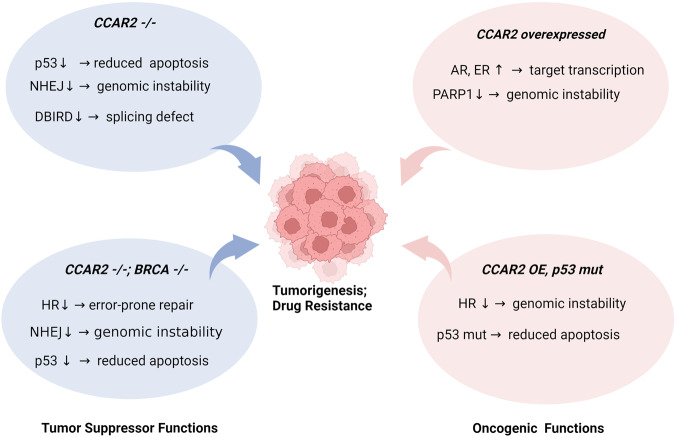
hCCAR2 status and tumorigenesis. **(Left)** hCCAR2 deletion is associated with decreased p53 expression, DSB repair (NHEJ), and splicing defects. In the absence of BRCA, genomic instability is further increased, yet affected cancer cells may be more refractory to chemotherapeutic drugs, possibly via activation of alternative repair pathways. (**Right**) Overexpression of hCCAR2 may increase the expression of certain oncogenic target genes while suppressing PARP1 to reduce the DNA repair capacity. In the p53 mutant background, reduced apoptosis may further drive tumorigenesis. Although it has not been experimentally demonstrated, overexpression of hCCAR2 may disrupt the balance between HR and NHEJ repair, increasing genomic instability.

Studies point to the identity of hCCAR2 as a tumor suppressor (Fig. 5) due to its role in inducing p53-mediated apoptosis and in decreasing utilization of the mutagenic NHEJ DNA repair pathway. Indeed, hCCAR2 is commonly mutated in breast and ovarian cancers^1^. Consistent with the idea that hCCAR2 is a tumor suppressor, CCAR2 (mCCAR2) knockout mice are prone to tumor development^45^; in addition, this tumor development induced by mCCAR2 deficiency was shown to be independent of SIRT1. Notably, hCCAR2 deficiency results in the destabilization of p53; hCCAR2 directly binds p53 and outcompetes mouse double minute 2 protein (MDM2), a ubiquitin ligase that mediates p53 degradation. This study implies that hCCAR2 can activate p53 in two different ways: by inhibiting SIRT1 (thus increasing p53 acetylation) and by directly stabilizing p53 (as shown in this study). However, this activity may also promote some cancers that are driven by mutant p53 proteins, as hCCAR2 can also stabilize mutant p53. The finding that CCAR2 knockout mice develop tumors is in line with the genome-stabilizing roles of CCAR2, such as its role in promoting DSB repair by balancing the use of HR and NHEJ^1^. CCAR2 may also exert tumor-suppressive effects by regulating transcription. hCCAR2 was shown to be a part of the messenger ribonucleoprotein (mRNP) complex (termed DBIRD^46^). Knocking down hCCAR2 disrupts RNA polymerase II (RNAPII) elongation and the splicing of a large set of exons. This hCCAR2-dependent transcriptional and splicing control may promote aberrant signaling events leading to cancer development.

The status of hCCAR2 may dictate cancer progression and drug resistance in the BRCA1/2-null background (Fig. 5). Consistent with experimental evidence that hCCAR2 knockout renders BRCA1-null cells resistant to DNA damaging agents, BRCA1-deficient breast cancer patients with a low hCCAR2 level have a worse prognosis than those with a high hCCAR2 level^1^. Thus, cancers with dual genetic loss of hCCAR2 and BRCA, in theory, are expected to be more aggressive and refractory to radio- or chemotherapeutic agents. These studies reinforce the idea that hCCAR2 acts as a tumor suppressor.

### Oncogenic roles

The findings indicating that hCCAR2 protein expression is elevated in some cancers suggest that hCCAR2 may also have oncogenic roles. hCCAR2 is overexpressed in gastric cancers, with an association with metastasis^47^, and high hCCAR2 expression is associated with unfavorable outcomes in hepatocarcinoma patients^48^. hCCAR2 is a coactivator of the androgen receptor (AR-V7 variant) and its downstream targets, such as CDH2, to increase the growth of prostate cancer cells^49^. hCCAR2 promotes the invasive properties of osteosarcoma cells, possibly by stabilizing AR proteins^50^. hCCAR2 associates with estrogen receptor α (Erα) to amplify the function of ERα, thus promoting the survival of MCF7 breast cancer cells^51^. hCCAR2 promotes the proliferation of squamous cell carcinoma (SCC), in part by promoting the stability of the oncogenic transcription factors regulatory factor X (RFX1) and cAMP responsive element binding protein 1 (CREB1)^52^. hCCAR2 binding and inhibition of PARP1^26^, a crucial regulator of various aspects of DNA repair, can potentially destabilize the genome and promote cancer development. Elevated hCCAR2 expression, through decreased SIRT1 activity and thus increased HSF1 activity, could also promote the adaptiveness of cancers to various stresses^20^. Thus, there are many mechanisms by which CCAR2 could function as an oncogene. In addition, when overexpressed, hCCAR2 may function as an oncogene by modulating the p53 status (Fig. 5). hCCAR2 stabilizes both wild-type and mutant p53^53^. Human cancers frequently express mutant p53, which inhibits wild-type p53 activity in a dominant-negative manner^54–56^. Thus, hCCAR2 overexpression in cancers with mutant p53 may foster tumorigenesis in conjunction with the other cancer-promoting effects of CCAR2 overexpression.

## hCCAR1 regulates cancer development, the cell cycle, transcription, and apoptosis

The roles of the hCCAR2 paralog hCCAR1 in the stress response, aging, and cancer development are less well characterized than those of hCCAR2, although several studies have shown that hCCAR1 regulates the cell cycle and apoptosis^57,58^. hCCAR1 was identified as an interactor of the anaphase-promoting complex (APC/C) E3 ligase, which regulates multiple steps during cell cycle transitions^59^. hCCAR1 may promote apoptotic signaling upon DNA damage by associating with and promoting the formation of phosphorylated H2A histone family member X (γH2AX)^60^.

In other studies, the hCCAR1 5’ UTR binds to the microRNA miR-1254 in a miRNA:mRNA host relationship^61^. MicroRNAs bind to miRNA binding sites in their target RNAs, leading to mutual regulation of expression. This miRNA:mRNA targeting relationship is mainly mediated by specific base-pairing interactions between the 5’ end of the miRNA (seed region) and the miRNA response elements within the coding region or untranslated region (UTR) of an mRNA, leading to mRNA destabilization/translational inhibition. This miRNA:mRNA regulatory relationship has been shown to regulate various processes, including cancer progression. hCCAR1 5’UTR binding to miRNA-1254 overcame resistance to tamoxifen, an estrogen receptor modulator used in ERα-positive breast cancer patients, in clinical trials^61^.

Studies have also found that hCCAR1 acts as a transcriptional coactivator. hCCAR1 is a participant in Wnt/beta catenin-dependent transcriptional signaling, and depletion of hCCAR1 inhibits the expression of several Wnt/beta-catenin target genes and suppresses anchorage-independent growth of colon cancer cells^62^. In response to hormones, hCCAR1 is recruited to nuclear receptor target genes and mediates the recruitment of the Mediator complex, which in turn recruits RNAPII^63^. Depletion of hCCAR1 inhibits hormone-induced recruitment of Mediator components and RNPAII to target gene promoters and thus suppresses estrogen-dependent growth of breast cancer cells. Thus, CCAR1 regulates the expression of key proliferation-inducing genes. This study also found that hCCAR1 is a coactivator of p53, suggesting its broader role in transcriptional regulation and implicating it as a tumor suppressor. hCCAR1 also promotes chromatin loading of the androgen receptor (AR) transcription complex^64^ and regulates glucocorticoid receptor-dependent transcription^65^. Thus, hCCAR1 impacts cancer development by acting as a coactivator of various transcriptional programs.

## Concluding remarks

The CCAR family members are evolutionarily conserved, contain several conserved domains, and interact with an increasing array of cellular networks. The CCAR proteins regulate processes, including stress responses, DNA damage and repair, apoptosis, transcription, splicing, oncogenesis, and aging. Some of the are mediated through the ability of CCAR to inhibit SIRT1, while others are independent of SIRT1. It is interesting that studies with CCAR-1 in *C. elegans* have implicated this protein as having a role in the healthspan and in aging. Whether this role is conserved in mammals remains to be seen.

In addition, whether CCAR2 loss shares other features of NHEJ deficiency awaits further investigation. Whether CCAR2’s role as part of the DBIRD complex contributes to cancer-promoting activities also awaits further investigation, given that roles of splicing factors in cancer development and genetic instability have emerged^66^.

How hCCAR2 mutations translate into pro- or anticancer effects remains enigmatic. It appears that the impact of the hCCAR2 status on tumorigenesis depends on the complexity of the genetic background of a cancer. While hCCAR2 deletion could compromise cancer cells’ invasive properties or resilience to various stresses, thus inducing cancer cell death, hCCAR2 deletion in an HR-deficient background (e.g., BRCA1/2 mutant) may confer a growth advantage on cancer cells by restoring genetic stability.

The hCCAR1 and hCCAR2 proteins are intrinsically disordered, a feature of proteins often found in membraneless organelles^2^. In addition to localizing to RNA stress granules in the cytosol, hCCAR2 undergoes phase separation into nuclear bodies (NBs) along with HNRNPL and RNA transcripts, as shown in a recent study showed that^67^. Although the biological roles of hCCAR2-containing NBs remain elusive, it is possible that some of the functions of nuclear hCCAR2 (e.g., directly binding to lncRNAs to regulate p53^46^) are regulated within, or by, these phase-separated compartments. Future studies will most likely identify additional functional roles for hCCAR proteins, both within and outside of the nucleus and both within and outside of membraneless organelles.

## References

[1] Iyer DR (2022). CCAR2 functions downstream of the Shieldin complex to promote double-strand break end-joining. Proc. Natl Acad. Sci. USA.

[2] Brunquell J, Yuan J, Erwin A, Westerheide SD, Xue B (2014). DBC1/CCAR2 and CCAR1 Are Largely Disordered Proteins that Have Evolved from One Common Ancestor. Biomed. Res Int.

[3] Rajendran P (2019). Acetylation of CCAR2 Establishes a BET/BRD9 Acetyl Switch in Response to Combined Deacetylase and Bromodomain Inhibition. Cancer Res.

[4] Zheng H (2013). hMOF acetylation of DBC1/CCAR2 prevents binding and inhibition of SirT1. Mol. Cell Biol..

[5] Kong S (2015). Deleted in Breast Cancer 1 Suppresses B Cell Activation through RelB and Is Regulated by IKKalpha Phosphorylation. J. Immunol..

[6] Anantharaman V, Aravind L (2008). Analysis of DBC1 and its homologs suggests a potential mechanism for regulation of sirtuin domain deacetylases by NAD metabolites. Cell Cycle.

[7] Chazin WJ (2011). Relating form and function of EF-hand calcium binding proteins. Acc. Chem. Res.

[8] Chini, E. N., Chini, C. C., Nin, V. & Escande, C. Deleted in breast cancer-1 (DBC-1) in the interface between metabolism, aging and cancer. *Biosci. Rep.* **33**. 10.1042/bsr20130062 (2013). 10.1042/BSR20130062 PMC3755336 23841676

[9] Chen C, Masi R, Lintermann R, Wirthmueller L (2018). Nuclear Import of Arabidopsis Poly(ADP-Ribose) Polymerase 2 Is Mediated by Importin-alpha and a Nuclear Localization Sequence Located Between the Predicted SAP Domains. Front Plant Sci..

[10] Hnizda A (2021). SAP domain forms a flexible part of DNA aperture in Ku70/80. FEBS J..

[11] Notenboom V (2007). Functional characterization of Rad18 domains for Rad6, ubiquitin, DNA binding and PCNA modification. Nucleic Acids Res.

[12] Kim HJ, Moon SJ, Hong S, Won HH, Kim JH (2022). DBC1 is a key positive regulator of enhancer epigenomic writers KMT2D and p300. Nucleic Acids Res.

[13] Kim JE, Chen J, Lou Z (2008). DBC1 is a negative regulator of SIRT1. Nature.

[14] Zhao W (2008). Negative regulation of the deacetylase SIRT1 by DBC1. Nature.

[15] Magni M (2015). CCAR2/DBC1 is required for Chk2-dependent KAP1 phosphorylation and repair of DNA damage. Oncotarget.

[16] Park JH (2014). Modification of DBC1 by SUMO2/3 is crucial for p53-mediated apoptosis in response to DNA damage. Nat. Commun..

[17] Gao J, Chen X, Gu Q, Liu X, Xu X (2016). SENP1-Mediated Desumoylation of DBC1 Inhibits Apoptosis Induced by High Glucose in Bovine Retinal Pericytes. J. Ophthalmol..

[18] Kang H (2011). Peptide switch is essential for Sirt1 deacetylase activity. Mol. Cell.

[19] Nin V (2012). Role of deleted in breast cancer 1 (DBC1) protein in SIRT1 deacetylase activation induced by protein kinase A and AMP-activated protein kinase. J. Biol. Chem..

[20] Raynes R (2013). The SIRT1 modulators AROS and DBC1 regulate HSF1 activity and the heat shock response. PLoS One.

[21] Brunquell J (2018). CCAR-1 is a negative regulator of the heat-shock response in Caenorhabditis elegans. Aging Cell.

[22] Kim W, Cheon MG, Kim JE (2017). Mitochondrial CCAR2/DBC1 is required for cell survival against rotenone-induced mitochondrial stress. Biochem Biophys. Res Commun..

[23] Kolobova E (2009). Microtubule-dependent association of AKAP350A and CCAR1 with RNA stress granules. Exp. Cell Res.

[24] Zannini L, Buscemi G, Kim JE, Fontanella E, Delia D (2012). DBC1 phosphorylation by ATM/ATR inhibits SIRT1 deacetylase in response to DNA damage. J. Mol. Cell Biol..

[25] Yuan J, Luo K, Liu T, Lou Z (2012). Regulation of SIRT1 activity by genotoxic stress. Genes Dev..

[26] Li J (2017). A conserved NAD(+) binding pocket that regulates protein-protein interactions during aging. Science.

[27] Eustermann S (2011). The DNA-binding domain of human PARP-1 interacts with DNA single-strand breaks as a monomer through its second zinc finger. J. Mol. Biol..

[28] Rajman L, Chwalek K, Sinclair DA (2018). Therapeutic Potential of NAD-Boosting Molecules: The In Vivo Evidence. Cell Metab..

[29] Magni M, Buscemi G, Zannini L (2018). Cell cycle and apoptosis regulator 2 at the interface between DNA damage response and cell physiology. Mutat. Res Rev. Mutat. Res.

[30] Kim JE, Lou Z, Chen J (2009). Interactions between DBC1 and SIRT 1 are deregulated in breast cancer cells. Cell Cycle.

[31] Ceccaldi R, Rondinelli B, D’Andrea AD (2016). Repair Pathway Choices and Consequences at the Double-Strand Break. Trends Cell Biol..

[32] Scully R, Panday A, Elango R, Willis NA (2019). DNA double-strand break repair-pathway choice in somatic mammalian cells. Nat. Rev. Mol. Cell Biol..

[33] Stinson BM, Loparo JJ (2021). Repair of DNA Double-Strand Breaks by the Nonhomologous End Joining Pathway. Annu Rev. Biochem.

[34] Dev H (2018). Shieldin complex promotes DNA end-joining and counters homologous recombination in BRCA1-null cells. Nat. Cell Biol..

[35] Ghezraoui H (2018). 53BP1 cooperation with the REV7-shieldin complex underpins DNA structure-specific NHEJ. Nature.

[36] Gupta R (2018). DNA Repair Network Analysis Reveals Shieldin as a Key Regulator of NHEJ and PARP Inhibitor Sensitivity. Cell.

[37] Mirman Z (2018). 53BP1-RIF1-shieldin counteracts DSB resection through CST- and Polalpha-dependent fill-in. Nature.

[38] Lopez-Saavedra A (2016). A genome-wide screening uncovers the role of CCAR2 as an antagonist of DNA end resection. Nat. Commun..

[39] Hiraike H (2010). Identification of DBC1 as a transcriptional repressor for BRCA1. Br. J. Cancer.

[40] Ayrapetov MK, Gursoy-Yuzugullu O, Xu C, Xu Y, Price BD (2014). DNA double-strand breaks promote methylation of histone H3 on lysine 9 and transient formation of repressive chromatin. Proc. Natl Acad. Sci. USA.

[41] Li Z (2009). Inhibition of SUV39H1 methyltransferase activity by DBC1. J. Biol. Chem..

[42] Chini CC, Escande C, Nin V, Chini EN (2010). HDAC3 is negatively regulated by the nuclear protein DBC1. J. Biol. Chem..

[43] Hamaguchi M (2002). DBC2, a candidate for a tumor suppressor gene involved in breast cancer. Proc. Natl Acad. Sci. USA.

[44] Johnson GS, Rajendran P, Dashwood RH (2020). CCAR1 and CCAR2 as gene chameleons with antagonistic duality: Preclinical, human translational, and mechanistic basis. Cancer Sci..

[45] Qin B (2015). DBC1 functions as a tumor suppressor by regulating p53 stability. Cell Rep..

[46] Close P (2012). DBIRD complex integrates alternative mRNA splicing with RNA polymerase II transcript elongation. Nature.

[47] Huan Y, Wu D, Zhou D, Sun B, Li G (2015). DBC1 promotes anoikis resistance of gastric cancer cells by regulating NF-kappaB activity. Oncol. Rep..

[48] Ha SY (2016). Expression of DBC1 is associated with poor prognosis in hepatitis virus-related hepatocellular carcinoma. Pathol. Res Pr..

[49] Moon SJ (2018). DBC1 promotes castration-resistant prostate cancer by positively regulating DNA binding and stability of AR-V7. Oncogene.

[50] Wagle S (2015). DBC1/CCAR2 is involved in the stabilization of androgen receptor and the progression of osteosarcoma. Sci. Rep..

[51] Yu EJ (2011). Reciprocal roles of DBC1 and SIRT1 in regulating estrogen receptor alpha activity and co-activator synergy. Nucleic Acids Res.

[52] Best SA, Nwaobasi AN, Schmults CD, Ramsey MR (2017). CCAR2 Is Required for Proliferation and Tumor Maintenance in Human Squamous Cell Carcinoma. J. Invest Dermatol.

[53] Akande OE (2019). DBC1 Regulates p53 Stability via Inhibition of CBP-Dependent p53 Polyubiquitination. Cell Rep..

[54] Hu J (2021). Targeting mutant p53 for cancer therapy: direct and indirect strategies. J. Hematol. Oncol..

[55] Tang Q (2021). Mutant p53 regulates Survivin to foster lung metastasis. Genes Dev..

[56] Zhu G (2020). Mutant p53 in Cancer Progression and Targeted Therapies. Front Oncol..

[57] Rishi AK (2003). Identification and characterization of a cell cycle and apoptosis regulatory protein-1 as a novel mediator of apoptosis signaling by retinoid CD437. J. Biol. Chem..

[58] Rishi AK (2006). Cell cycle- and apoptosis-regulatory protein-1 is involved in apoptosis signaling by epidermal growth factor receptor. J. Biol. Chem..

[59] Puliyappadamba VT (2011). Antagonists of anaphase-promoting complex (APC)-2-cell cycle and apoptosis regulatory protein (CARP)-1 interaction are novel regulators of cell growth and apoptosis. J. Biol. Chem..

[60] Sekhar S. C. et al. A H2AX(-)CARP-1 Interaction Regulates Apoptosis Signaling Following DNA Damage. *Cancers (Basel)* **11**, 10.3390/cancers11020221 (2019). 10.3390/cancers11020221 PMC6406907 30769864

[61] Li G (2016). CCAR1 5’ UTR as a natural miRancer of miR-1254 overrides tamoxifen resistance. Cell Res.

[62] Ou CY, Kim JH, Yang CK, Stallcup MR (2009). Requirement of cell cycle and apoptosis regulator 1 for target gene activation by Wnt and beta-catenin and for anchorage-independent growth of human colon carcinoma cells. J. Biol. Chem..

[63] Kim JH (2008). CCAR1, a key regulator of mediator complex recruitment to nuclear receptor transcription complexes. Mol. Cell.

[64] Seo WY (2013). CCAR1 promotes chromatin loading of androgen receptor (AR) transcription complex by stabilizing the association between AR and GATA2. Nucleic Acids Res.

[65] Ou CY, Chen TC, Lee JV, Wang JC, Stallcup MR (2014). Coregulator cell cycle and apoptosis regulator 1 (CCAR1) positively regulates adipocyte differentiation through the glucocorticoid signaling pathway. J. Biol. Chem..

[66] Oltean S, Bates DO (2014). Hallmarks of alternative splicing in cancer. Oncogene.

[67] Mannen T (2021). Distinct RNA polymerase transcripts direct the assembly of phase-separated DBC1 nuclear bodies in different cell lines. Mol. Biol. Cell.

